# Evaluation of effectiveness and safety of hydroxyethyl starch (HES 130 kDa/0.4) in burn resuscitation

**DOI:** 10.1186/cc10859

**Published:** 2012-03-20

**Authors:** A Mokline, I Rahmani, L Gharsallah, H Oueslati, B Gasri, I Harzallah, A Ksontini, A Messadi

**Affiliations:** 1 Burn and Trauma Center, Tunis, Tunisia

## Introduction

Excessive fluid resuscitation of large burn injuries has been associated with adverse outcomes including worsening of burn oedema, conversion of superficial into deep burns, and compartment syndromes. So, there have been efforts recently to address these concerns, particularly with the use of physiologically balanced fluids. Starches, as effective plasma expanders, may limit resuscitation requirements and burn oedema. This study aims to evaluate clinical results of HES in early burn resuscitation of major burn-injured patients.

## Methods

A case-control study conducted in a burn care center in Tunis. Adult burned patients admitted within the first 24 hours post burn, with a burn injury exceeding 30% of total body surface area, from 1 January to 31 December 2010 were included. Exclusion criteria were pregnancy, history or biochemical evidence of renal impairment on admission (serum creatinine >130 μmol/l), history or hematological evidence of disorders of hemostasis. Fluid volume resuscitation was evaluated according to the Parkland formula. HES supplementation was limited to 33 ml/kg/24 hours. The HES supplementation group was compared with a group of patients from the same center matched in age, sex and severity of burns at baseline.

## Results

Patients were assigned to two groups: G1 (*n *= 15): HES supplemented, and G2 (*n *= 15): crystalloids only. The mean age was 44 ± 18 years old for G1 and 43 ± 17 years old for G2. The average TBSA was 51.8% ± 19 for G1 versus 43.6 ± 7 for G2. The addition of HES 130 kDa/0.4 reduces significantly body weight gain within the first 72 hours after injury: 8 kg for G1 versus 13.6 kg for G2 (*P *= 0.002), occurrence of ALI (35% for G1 versus 65% for G2) (*P *= 0.01), and length of ICU stay (19 days ± 13 for G1 vs. 30 days ± 15 for G2). There was no evidence of renal dysfunction with the use of HES in burns patients comparative to the crystalloids group.

## Conclusion

HES supplementation in early burn resuscitation allows, for smaller fluid volume requirement, less tissue oedema. This along with a significantly lower in ALI occurrence and length of ICU stay.

